# Aerobic Exercise-Based Pulmonary Rehabilitation for Bronchial Asthma in Pediatrics: A Systematic Review

**DOI:** 10.7759/cureus.96568

**Published:** 2025-11-11

**Authors:** Salma Eltohami Ballal Tahaelbashir, Wiaam Noureldin Birima Noureldin, Islam Kanada Toto Korea, Rayan Elnoor Saad Mohamed, Asma Abualgasim Alameen Ahmed, Mohamed Alshaab M Alshibane

**Affiliations:** 1 Paediatrics, Armed Forces Hospitals, Southern Region, Khamis Mushait, SAU; 2 Pediatric Emergency, Bristol Children Hospital NHS, Bristol, GBR; 3 General Practice, University of Medical Sciences and Technology, Khartoum, SDN; 4 Pediatrics, Sudan Medical Specialization Board, Khartoum, SDN; 5 Pediatrics, Ministry of Health, Almadinah Almonwarra, SAU; 6 Biomedical Sciences, University of Reading, Reading, GBR

**Keywords:** adolescent, aerobic exercise, asthma, child, pediatrics, physical activity, pulmonary rehabilitation, quality of life, systematic review

## Abstract

Bronchial asthma is a prevalent chronic condition in children, often leading to exercise intolerance and diminished quality of life despite pharmacological management. Aerobic exercise-based pulmonary rehabilitation (PR) has emerged as a promising adjunct therapy. This systematic review aims to critically evaluate the effects of aerobic exercise-based PR on pulmonary function, asthma control, exercise capacity, and quality of life in children with bronchial asthma. A systematic search was conducted across five electronic databases (PubMed, Scopus, Embase, Web of Science, and ClinicalTrials) for randomized controlled trials (RCTs) published from 2015 to 2025. Studies involving children (≤18 years) with asthma that compared aerobic exercise-based interventions against a control were included. Two independent reviewers performed study selection, data extraction, and risk of bias assessment using the Cochrane Risk of Bias 2 (ROB 2) tool. A narrative synthesis was undertaken due to heterogeneity in interventions and outcomes. Eight RCTs with a total of 435 participants were included. The findings revealed inconsistent effects on spirometric parameters [forced expiratory volume in one second (FEV₁), forced vital capacity (FVC)], with three studies showing significant improvements and others reporting no significant change. In contrast, interventions consistently demonstrated significant benefits in asthma control/symptoms, quality of life, and cardiorespiratory fitness (e.g., VO₂max, 6-minute walk test). Various modalities, including yoga, conventional aerobic exercise, and inspiratory muscle training, were effective. The overall risk of bias was low for seven of the eight included studies. Aerobic exercise-based pulmonary rehabilitation is a safe and effective adjunct to standard care in pediatric asthma, conferring significant and consistent improvements in asthma control, quality of life, and physical fitness, even in the absence of uniform changes in lung function. These findings support the integration of structured exercise programs into comprehensive asthma management plans for children. However, the heterogeneity in exercise interventions and outcome measurements across the included studies should be considered when interpreting these findings.

## Introduction and background

Bronchial asthma is one of the most prevalent chronic respiratory conditions affecting children worldwide, characterized by airway inflammation, hyperresponsiveness, and episodic airflow obstruction [1]. It significantly impairs the quality of life of affected children by limiting physical activity, school participation, and social engagement, while imposing a substantial economic and clinical burden on healthcare systems [2]. Although pharmacological management-primarily involving inhaled corticosteroids, bronchodilators, and leukotriene receptor antagonists-remains the cornerstone of treatment, a considerable proportion of pediatric patients continue to experience suboptimal control, recurrent symptoms, and reduced exercise tolerance despite adherence to medication [3]. This persistent functional limitation highlights the need for complementary non-pharmacological strategies that address the broader physiological and lifestyle dimensions of asthma care.

In this context, aerobic exercise-based pulmonary rehabilitation (PR) has gained attention as a potential adjunctive approach to traditional asthma management [4]. Aerobic exercise interventions, including swimming, running, cycling, and structured aerobic training, have demonstrated physiological benefits such as enhanced cardiorespiratory endurance, improved respiratory muscle performance, and reduced airway inflammation [5]. By integrating aerobic training into PR, these programs aim to strengthen pulmonary function, improve asthma control, and promote sustained physical activity, thereby supporting both short- and long-term disease management [6].

However, existing research presents inconsistent findings regarding the magnitude and clinical significance of these benefits in pediatric populations. Variations in study design, exercise type, duration, and intensity contribute to heterogeneous outcomes, with some trials reporting significant improvements in forced expiratory volume in one second (FEV₁), peak expiratory flow rate (PEFR), and symptom control, while others show minimal or inconclusive effects [4, 7, 8]. Moreover, evidence concerning the safety, feasibility, and sustainability of aerobic exercise-based pulmonary rehabilitation (PR) across different severities of childhood asthma remains limited.

This gap in synthesized knowledge underscores the need for a comprehensive and methodologically rigorous review of current evidence. Therefore, this systematic review aims to critically evaluate randomized controlled trials investigating the effects of aerobic exercise-based pulmonary rehabilitation on clinical and functional outcomes in children with bronchial asthma. By addressing this gap, the review seeks to clarify the efficacy and practical implications of exercise-based interventions and provide evidence-based guidance for clinicians, caregivers, and researchers in optimizing pediatric asthma management.

## Review

Methods

Eligibility Criteria

This systematic review included randomized controlled trials (RCTs) assessing the effects of aerobic exercise-based pulmonary rehabilitation in children diagnosed with bronchial asthma. RCTs were specifically selected because they provide the highest level of evidence for intervention efficacy, minimizing confounding factors and bias compared to observational studies. To ensure the inclusion of the most up-to-date evidence, only studies published between 2015 and 2025 were considered. Both full-text publications and studies identified through the reference lists of relevant articles were included to capture all pertinent data. Studies were excluded if they involved non-aerobic interventions, mixed adult and pediatric populations without separate pediatric data, or lacked clinical or functional outcome measures.

The inclusion and exclusion criteria are summarized according to the population, intervention, comparison, outcomes, and study (PICOS) framework [9] in Table 1.

**Table 1:** Eligibility Criteria for Studies Inclusion Eligibility Criteria for Studies Inclusion according to the PICOS framework [9]. FEV₁: forced expiratory volume in one second, FVC: forced vital capacity; PEFR: peak expiratory flow rate. (PEFR); ACT: asthma control test.

PICOS	Criteria
Population	Children (≤18 years) diagnosed with bronchial asthma
Intervention	Aerobic exercise-based pulmonary rehabilitation programs (e.g., swimming, cycling, treadmill training)
Comparator	Standard care, placebo, or non-exercise control
Outcomes	Pulmonary function (FEV₁, FVC, PEFR), asthma control measures (e.g., ACT), quality of life, exercise capacity
Study Design	Randomized controlled trials (RCTs)

Information Sources and Search Strategy

A comprehensive literature search was conducted in PubMed, Scopus, Embase, Web of Science, and ClinicalTrials to identify eligible studies. The search strategy combined keywords and medical subject headings (MeSH) related to “pediatric asthma,” “aerobic exercise,” and “pulmonary rehabilitation.” To ensure thorough coverage, backward citation tracking of included studies and relevant reviews was also performed. Only articles published in English between January 2015 and May 2025 were considered. The detailed search strategy for each database is provided in the Appendix.

Study Selection

All identified records were imported into EndNote X9 (Clarivate, Philadelphia, USA) to remove duplicates. Two independent reviewers screened titles and abstracts for relevance, followed by a full-text review of potentially eligible studies. Any discrepancies between reviewers were resolved through discussion or consultation with a third reviewer. This rigorous selection process ensured that only studies meeting the predefined PICOS criteria were included.

Data Collection Process

Data extraction was performed independently by two reviewers using a standardized form. Extracted data included study characteristics (author, year, country), participant details (age, sample size, asthma severity), intervention details (type, intensity, frequency, and duration of aerobic exercise), comparator, outcome measures, and follow-up duration. Cross-checking of data ensured accuracy and completeness.

Risk of Bias Assessment

The quality of included RCTs was assessed using the Cochrane Risk of Bias tool version 2 (RoB 2) [10]. This tool evaluates potential biases in randomization, deviations from intended interventions, missing outcome data, measurement of outcomes, and selection of reported results. Studies were categorized as low risk, some concerns, or high risk of bias, and assessments were independently conducted by two reviewers with disagreements resolved through discussion.

Data Synthesis

Due to substantial heterogeneity in intervention types, exercise protocols, outcome measures, and follow-up durations, a meta-analysis was not performed. Heterogeneity was assessed through visual and descriptive comparison of study characteristics, including variations in participant demographics, intervention intensity and duration, and outcome reporting. The considerable methodological differences and variability in reported results precluded meaningful statistical pooling; therefore, a narrative synthesis was deemed the most appropriate approach to summarize the current evidence. Studies were systematically described in terms of participant characteristics, intervention protocols, and clinical and functional outcomes, highlighting emerging trends and inconsistencies.

Results

Study Selection Process

**Figure 1:**
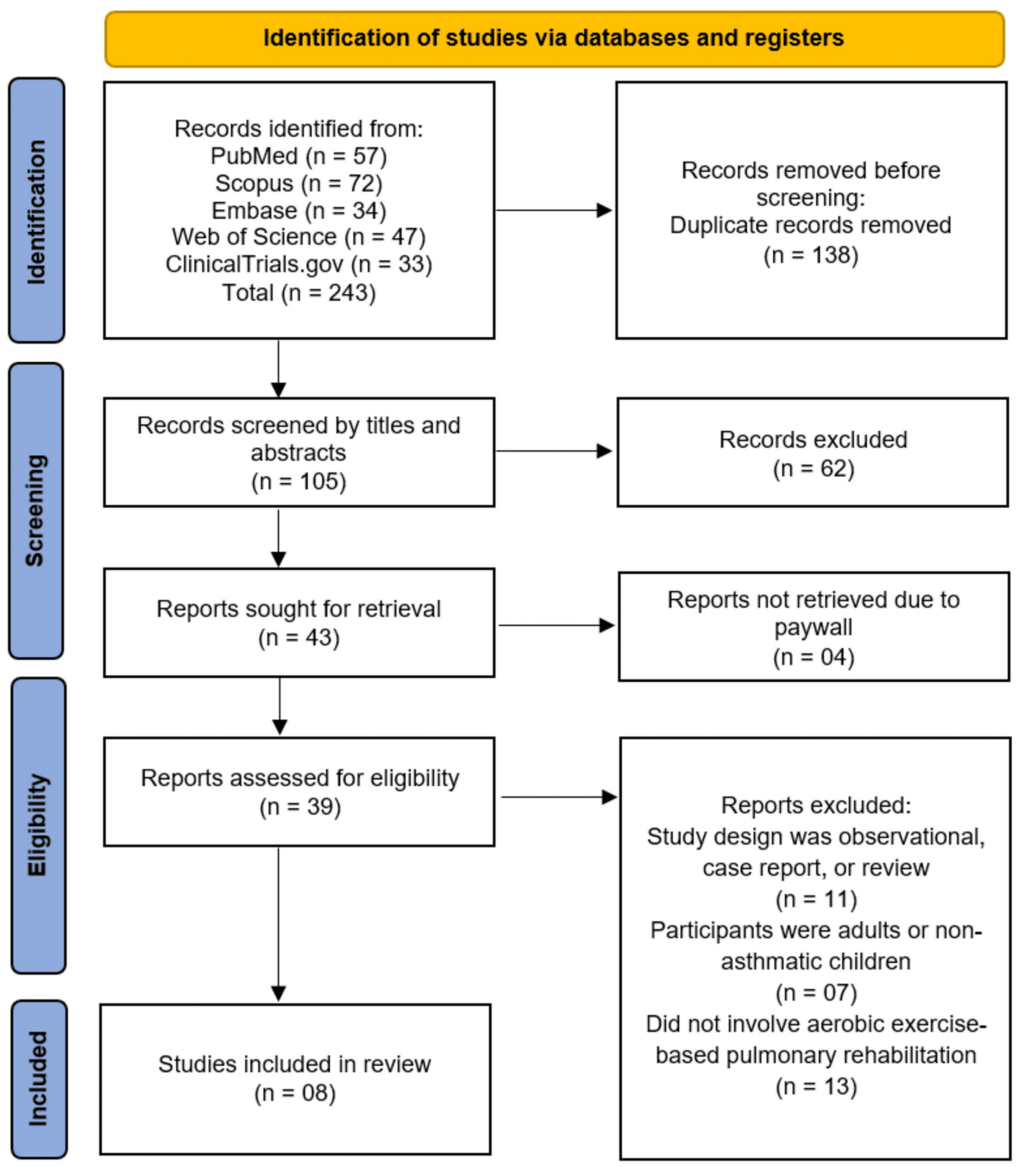
PRISMA Flowchart

The systematic search across five databases and registers (PubMed, Scopus, Embase, Web of Science, and ClinicalTrials) initially identified 243 records. After the removal of 138 duplicate records, a total of 105 unique studies were screened based on their titles and abstracts. This screening phase led to the exclusion of 62 records that did not meet the preliminary inclusion criteria. The full texts of the remaining 43 articles were sought for retrieval, of which four could not be accessed. Consequently, 39 full-text articles were assessed for eligibility. Upon detailed evaluation, 31 articles were excluded for the following reasons: the study design was observational, a case report, or a review (n=11); the participants were adults or non-asthmatic children (n=7); or the intervention did not involve an aerobic exercise-based pulmonary rehabilitation component (n=13). This rigorous selection process culminated in the inclusion of 8 studies [11-18] that satisfied all the predefined criteria for this systematic review (Figure 1).

Study Characteristics

A total of eight RCTs [11-18] were included in this systematic review, investigating the effects of various aerobic exercise-based pulmonary rehabilitation interventions in pediatric populations with asthma. The characteristics of these studies are summarized in Table 2. The included studies were conducted across diverse geographical regions, including China [11, 18], the USA [12], India [13], Spain [14], Egypt [15], Iran [16], and Saudi Arabia [17]. Sample sizes ranged from 15 [16] to 140 [13] participants, with the age of participants spanning from young children (four years) [11] to adolescents [12].

**Table 2:** Characteristics of Included Randomized Controlled Trials ET: exercise training; FEV₁: forced expiratory volume in one second; FVC: forced vital capacity; PADQLQ: pediatric asthma daily quality of life questionnaire; PEFR: peak expiratory flow rate; PAQLQ: pediatric asthma quality of life questionnaire; IG: intervention group; CG: control group; MWT: minute walk test; PQoLQ: pediatric quality of life questionnaire; IMT: inspiratory muscle training; IPmax: maximal inspiratory pressure; CRR: conventional rehabilitation regimen; EPmax: maximal expiratory pressure; ACT: asthma control test.

Author (Year)	Country / Region	Sample Size (n)	Age Range (Years)	Asthma Severity / Diagnostic Criteria	Intervention (Aerobic Exercise Type, Frequency, Intensity, Duration)	Control Group	Outcome Measures	Follow-up Duration
Zhang et al. [11]	China	72 (36 intervention, 36 control)	4–12	Mild asthma	ET + Montelukast; details on aerobic exercise type, frequency, and intensity not specified; duration: 6 weeks	Montelukast alone	Lung function (FEV₁, FEV₁/FVC), Clinical symptom score, Quality of life (PADQLQ), Adverse events	6 weeks treatment + 2 weeks follow-up
Bignall et al. [12]	USA (Urban, African-American adolescents)	33	Adolescents	Physician-diagnosed asthma	Breathing retraining (20-minute session) + asthma education; school-based	Standard asthma education (20-minute session)	Asthma control, Asthma Quality of Life, Lung Function (FEV₁, Peak Flow), State and Trait Anxiety	1 month
Yadav et al. [13]	North India	140 (70 intervention, 70 control)	10–16 years	Newly diagnosed bronchial asthma	Yoga-based aerobic exercise, supervised sessions; frequency and intensity not specified; duration: 3 months	Pharmacological treatment only	FVC, FEV₁, FEV₁/FVC, PEFR, and PAQLQ scores (activity, symptoms, emotional function)	12 weeks (3 months)
Sanz‐Santiago et al. [14]	Spain	53 (IG=25, CG=28)	11.5 ± 2.6	Mild–moderate asthma with exercise-induced symptoms	Combined aerobic and resistance exercise; 3 days/week; 60 minutes/session	Routine clinical orientations	Cardiorespiratory fitness, muscle strength, lung function, asthma control, quality of life, functional tests	12 weeks
Abdelbasset et al. [15]	Egypt	38	8–12	School-aged children with asthma	Moderate-intensity aerobic exercise; 10 weeks; frequency not explicitly mentioned; combined with asthma medications	Conventional treatment group: asthma medications only; home respiratory exercises recommended for both groups	Pulmonary function tests, VO₂max, 6MWT, fatigue index, PQoLQ	10 weeks
Khodashenas et al. [16]	Iran	15	6–18	Asthmatic children	Aerobic exercise + strength training; 45-min sessions, 3 times/week, for 8 weeks	Routine pharmacotherapy only	Spirometry (FEV1, FVC, FEF25-75%), Motor competency (Ozeretski test), Quality of life (St. George’s questionnaire)	8 weeks
Elnaggar [17]	Saudi Arabia	34 (IMT: 17, Placebo: 17)	Not specified	Children with asthma	IMT at 40% of maximal IPmax, 20 min/session, 3 times/week, 12 weeks, combined with CRR	Placebo IMT at 5% IPmax + CRR	Respiratory functions (FEV1, FVC, FEV1/FVC), respiratory muscle strength (IPmax, EPmax), ACT	12 weeks
Yang et al. [18]	China (Chongqing)	50	Not specified	Children with asthma	Combined respiratory muscle and exercise training (details on type, frequency, intensity, duration not specified) + routine drug treatment and health education	Routine drug treatment and health education	Inspiratory muscle strength (maximum inspiratory pressure), exercise capacity (6-minute walk test), spirometry measurements, asthma control, quality-of-life	3 months

The severity of asthma among participants varied, encompassing mild [11], mild-to-moderate with exercise-induced symptoms [14], and physician-diagnosed asthma without further specification in some studies [12, 13]. The interventions were heterogeneous in their design. While all incorporated a component of physical training, the specific modalities included general aerobic exercise training [11, 15], yoga [13], combined aerobic and resistance training [14, 16], breathing retraining [12], and inspiratory muscle training (IMT) [17, 18]. The intervention duration also varied considerably, from four weeks [12] to 12 weeks [13, 14, 17, 18]. Control groups typically received standard pharmacological care alone [11, 13, 15, 16] or in combination with health education [12, 18] or placebo training [17].

Pulmonary Function Outcomes

The impact of exercise interventions on pulmonary function, as measured by spirometric parameters, was mixed across the included studies. Table 3 provides a detailed overview of these clinical and functional outcomes.

**Table 3:** Clinical and Functional Outcomes from Included RCTs Included in this Systematic Review FEV₁: forced expiratory volume in one second; FVC: forced vital capacity; PEFR: peak expiratory flow rate; ACT: asthma control test; MWT: minute walk test; PAQLQ: pediatric asthma quality of life questionnaire; AQLQ: asthma quality of life questionnaire; NR: not reported; PADQLQ: pediatric asthma daily quality of life questionnaire; ET: exercise training; AE: Aerobic Exercise; PQoL: pediatric quality of life; ttt: treatment; QoL: quality of life; FEF: forced expiratory flow; IMT: inspiratory muscle training, CRR: conventional rehabilitation regimen; NS: non significant.

Author (Year)	Change in FEV₁ (% Predicted)	Change in FVC (% Predicted)	Change in PEFR (L/min)	Change in ACT/Asthma Control	Change in Exercise Capacity (VO₂max / 6MWT)	Change in Quality of Life (PAQLQ/AQLQ)	Key Finding
Zhang et al. [11]	No significant improvement vs control (p>0.05)	No significant difference (FEV₁/FVC, p>0.05)	NR	Significant improvement in clinical symptoms (p<0.01)	NR	Significant improvement in PADQLQ scores (p<0.01)	ET plus montelukast improved asthma symptoms and quality of life, but not lung function, compared to montelukast alone.
Bignall et al. [12]	No significant change	NR	No significant change	Significant improvement over time (p≤0.01), but no difference between groups	NR	Significant improvement over time (p≤0.01), but no difference between groups	Breathing retraining was feasible and acceptable; it improved asthma control and quality of life over time, but no between-group differences in lung function or control outcomes.
Yadav et al. [13]	Significant improvement in FEV₁ compared to the control group	Significant improvement in FVC compared to the control group	Significant improvement in PEFR compared to the control group	Overall, asthma control improved	NR	Significant improvement in PAQLQ total and domain scores	Yoga as an adjunct to pharmacological therapy significantly improved pulmonary function and quality of life compared to pharmacological therapy alone
Sanz‐Santiago et al. [14]	No significant change	No significant change	NR	No significant change	↑ Peak VO₂ (p=0.008), ↑ ventilatory threshold (p=0.025), ↑ test duration (p=0.014)	No significant change	Combined aerobic + resistance training improved cardiorespiratory fitness and muscle strength but did not significantly change lung function, asthma control, or quality of life.
Abdelbasset et al. [15]	Significant improvement in AE group; higher than Con ttt (p<0.05)	Significant improvement in AE group; higher than Con ttt (p<0.05)	NR	NR	VO₂max improved significantly in AE group (p<0.05); 6MWT improved in AE group (p<0.05); fatigue index improved in AE group (p<0.05)	All dimensions of PQoL significantly improved in the AE group (p<0.05); no significant improvement in the Con ttt group	Moderate-intensity aerobic exercise for 10 weeks improved pulmonary function, exercise capacity, and pediatric quality of life more than conventional treatment alone.
Khodashenas et al. [16]	Improved	Improved	NR	NR	NR	Improved (St. George's QoL questionnaire, significant)	Aerobic exercise significantly improved spirometric parameters (FEF, FEF25-75%), quality of life, and motor competency in asthmatic children. Aerobic exercise and strength training are useful complementary treatments.
Elnaggar [17]	↑ FEV₁ (p=.003)	↑ FVC (p=.001)	NR	↑ ACT (P=.001)	NR	NR	IMT + CRR improved respiratory function, muscle strength, and asthma symptom perception in children with asthma
Yang et al. [18]	NS (p>0.05)	NS (p>0.05)	NS (p>0.05)	↑ (p<0.05)	NS (p>0.05)	↑ (p<0.05)	Combined respiratory muscle + exercise training improved asthma control and quality of life, but not spirometry or exercise capacity

Three studies reported significant improvements in forced expiratory volume in 1 second (FEV₁) and forced vital capacity (FVC) in the intervention groups compared to the control groups. Yadav et al. [13] found that yoga-based aerobic exercise led to significant improvements in FEV₁, FVC, and PEFR. Similarly, Abdelbasset et al. [15] demonstrated that a 10-week moderate-intensity aerobic exercise program resulted in significant improvements in FEV₁ and FVC. Khodashenas et al. [16] also reported improved spirometric parameters, including FEV₁ and FVC, following an eight-week program of aerobic and strength training.

In contrast, several other studies found no significant between-group differences in these standard lung function measures. Zhang et al. [11] and Sanz-Santiago et al. [14] reported no significant improvements in FEV₁ or FVC. Bignall et al. [12] also found no significant change in FEV₁ or peak flow following breathing retraining. Similarly, Yang et al. [18] reported no significant changes in FEV₁, FVC, or PEFR after combined respiratory muscle and exercise training. One study focusing on IMT showed a different pattern, with Elnaggar [16] reporting significant increases in both FEV₁ and FVC following inspiratory muscle training.

Asthma Control and Symptom Outcomes

The effect of exercise interventions on asthma control and clinical symptoms was more consistently positive. Multiple studies demonstrated significant benefits. Zhang et al. [12] reported a significant improvement in clinical symptom scores when exercise training was combined with montelukast. Bignall et al. [12] observed a significant improvement in asthma control over time in their breathing retraining group, although no significant difference was found between the intervention and control groups. Yadav et al. [13] noted an overall improvement in asthma control with yoga therapy.

Studies employing IMT showed particularly strong results in this domain. Elnaggar [17] found a significant increase in ACT scores following IMT, and Yang et al. [18] also reported a significant improvement in asthma control despite the lack of change in spirometry. Conversely, Sanz-Santiago et al. [14] found no significant change in asthma control following their combined exercise program.

Exercise Capacity and Cardiorespiratory Fitness

Improvements in exercise capacity and cardiorespiratory fitness were observed in several studies. Sanz-Santiago et al. [14] reported that their combined aerobic and resistance program significantly improved peak VO₂, ventilatory threshold, and test duration, indicating enhanced cardiorespiratory fitness. Abdelbasset et al. [15] found that their aerobic exercise program led to a significant increase in VO₂max and performance in the six-minute walk test (6MWT), alongside a reduction in fatigue index. However, Yang et al. [18] did not find a significant improvement in the 6MWT following their combined training intervention.

Quality of Life Outcomes

A majority of the studies that assessed QoL reported significant improvements in the intervention groups. Significant enhancements in QoL scores, measured using various questionnaires such as the Pediatric Asthma Quality of Life Questionnaire (PAQLQ), were documented by Zhang et al. [11], Yadav et al. [13], and Abdelbasset et al. [15]. Bignall et al. [12] also noted a significant improvement in asthma-related quality of life over time. Khodashenas et al. [16] and Yang et al. [18] similarly reported significant improvements in QoL following their respective exercise interventions. In contrast, Sanz-Santiago et al. [14] was the only study among those measuring QoL that found no significant change.

Summary of Key Findings

In synthesis, the findings from the eight included RCTs indicate that aerobic exercise-based pulmonary rehabilitation is a safe and feasible adjunct to standard pharmacological therapy in pediatric asthma. The most consistent benefits were observed in domains of asthma control, quality of life, and cardiorespiratory fitness. Improvements in conventional spirometric parameters (FEV₁, FVC) were less consistent, suggesting that the clinical benefits of exercise may extend beyond direct improvements in airway obstruction as measured by standard lung function tests. Interventions such as yoga [13], moderate-intensity aerobic exercise [15], and inspiratory muscle training [17] demonstrated particularly positive outcomes across multiple domains.

Risk of Bias in Included Studies

The assessment revealed that the majority of studies were judged to have a low risk of bias across all domains, including the randomization process, deviations from intended interventions, missing outcome data, measurement of the outcome, and selection of the reported result [11-16, 18]. However, one study by Elnaggar [17] was rated with a high overall risk of bias; this judgment was primarily due to a high risk of bias in the measurement of the outcome, as the lack of blinding for participant-reported and effort-dependent outcomes was deemed likely to significantly influence the results, alongside some concerns regarding the selection of the reported result. Consequently, the body of evidence synthesized in this review is predominantly comprised of studies with a low risk of bias (Table 4).

**Table 4:** Risk of Bias Assessment using the Cochrane ROB 2 Tool

Study (Author, Year)	Domain 1: Randomization Process	Domain 2: Deviations from Intended Interventions	Domain 3: Missing Outcome Data	Domain 4: Measurement of the Outcome	Domain 5: Selection of the Reported Result	Overall Risk of Bias
Zhang et al. [11]	Low risk	Low risk	Low risk	Low risk	Low risk	Low risk
Bignall et al. [12]	Low risk	Low risk	Low risk	Low risk	Low risk	Low risk
Yadav et al. [13]	Low risk	Low risk	Low risk	Low risk	Low risk	Low risk
Sanz‐Santiago et al. [14]	Low risk	Low risk	Low risk	Low risk	Low risk	Low risk
Abdelbasset et al. [15]	Low risk	Low risk	Low risk	Low risk	Low risk	Low risk
Khodashenas et al. [16]	Low risk	Low risk	Low risk	Low risk	Low risk	Low risk
Elnaggar [17]	Low risk	Low risk	Low risk	High risk	Some concerns	High risk
Yang et al. [18]	Low risk	Low risk	Low risk	Low risk	Low risk	Low risk

Discussion

This systematic review aimed to synthesize the evidence from eight RCTs [11-18] investigating the efficacy of aerobic exercise-based pulmonary rehabilitation for bronchial asthma in the pediatric population. The collective findings from these diverse studies, conducted across multiple continents, paint a compelling picture: while the effect of exercise on conventional spirometric measures is inconsistent, its role as a beneficial adjunct to standard pharmacological therapy is strongly supported by consistent and clinically meaningful improvements in asthma control, quality of life, and cardiorespiratory fitness. This dichotomy suggests that the therapeutic value of exercise in pediatric asthma extends beyond simply modifying airway physiology, touching upon broader aspects of the disease experience and physical conditioning that are crucial for a child's overall well-being.

The most nuanced finding of this review pertains to pulmonary function. The results were heterogeneous, with three studies demonstrating significant improvements in FEV₁ and FVC [13, 15, 16], while an equal number found no significant between-group differences [11, 14, 18]. This inconsistency is a critical point of interpretation. It is plausible that the interventions that improved spirometry, such as the yoga program by Yadav et al. [13] and the moderate-intensity aerobic training by Abdelbasset et al. [15], were of sufficient duration, intensity, or specific type to elicit direct effects on airway smooth muscle and inflammation. Yoga, in particular, incorporates controlled breathing (pranayama), which may directly improve bronchial hyperresponsiveness and lung mechanics. However, the null findings from other high-quality interventions, such as the combined aerobic and resistance program by Sanz-Santiago et al. [14], indicate that improved lung function, as measured by FEV₁ and FVC, is not a guaranteed outcome. This aligns with the pathophysiological understanding that exercise may not reverse the underlying airway remodeling in all asthmatic children but can profoundly impact how they perceive and cope with their disease. Our findings are consistent with a meta-analysis by Wanrooij et al. [19], which concluded that physical training in asthmatic children significantly improved clinical outcomes without consistently altering lung function, suggesting that the benefits operate through alternative pathways.

Where the evidence becomes far more consistent and compelling is in the domain of asthma control and symptom perception. A majority of the included studies reported significant improvements in either clinical symptom scores or standardized asthma control questionnaires [11-13, 17, 18]. The study by Zhang et al. [11] found that exercise training added to montelukast led to better symptom control than medication alone. More strikingly, the studies focusing on respiratory muscle training, particularly the rigorous placebo-controlled trial by Elnaggar [17], demonstrated robust improvements in ACT scores. This suggests that interventions targeting the respiratory musculature may enhance neurorespiratory drive and reduce the perception of dyspnea, a common and distressing symptom in asthma. This reduction in symptom burden and improved sense of control is a fundamental goal of asthma management. These results corroborate the work of Baldwin et al. [20], who found that breathing exercises improved symptoms and quality of life in adults with asthma, and our review extends this evidence to the pediatric population, highlighting its feasibility even in school-based settings, as shown by Bignall et al. [12].

Perhaps the most patient-centric and significant outcome consistently favored the exercise interventions: health-related QoL. Six of the seven studies that measured QoL documented significant improvements [11-13, 15, 16, 18]. This is a powerful finding, as QoL encompasses the functional, emotional, and social impact of the disease from the child's perspective. The improvements reported across various validated questionnaires, such as the PAQLQ, indicate that children participating in these programs felt less limited in their activities, experienced fewer symptoms, and had improved emotional well-being. This can be attributed to a combination of factors, including increased self-efficacy, reduced fear of exercise-induced symptoms, and improved physical capacity, breaking the vicious cycle of inactivity and deconditioning common in asthma. The profound impact on QoL seen in our review is strongly supported by the findings of Carson et al. [21], who demonstrated that physical activity interventions led to significant improvements in asthma-related quality of life, underscoring the importance of moving beyond purely physiological endpoints.

The third pillar of benefit established by this review is the enhancement of exercise capacity and cardiorespiratory fitness. Studies by Sanz-Santiago et al. [14] and Abdelbasset et al. [15] provided objective evidence of this, showing significant increases in VO₂max, ventilatory threshold, and 6-minute walk test performance. This is a crucial outcome, as exercise-induced bronchoconstriction is a major barrier to physical activity for asthmatic children, often leading to a sedentary lifestyle and subsequent cardiovascular deconditioning. By systematically and safely exposing children to graded exercise, these rehabilitation programs appear to improve their physiological capacity to perform physical work, potentially by improving mechanical efficiency, increasing anaerobic threshold, and reducing the ventilatory demand for a given workload. This finding is in direct agreement with a systematic review by Wu et al. [22], which concluded that aerobic training improves exercise capacity in children and adolescents with asthma. Furthermore, the improvement in fitness itself may contribute to better asthma control, as proposed by Janssen et al. [23], who found an association between higher levels of physical activity and better asthma control in children.

When considering the various modalities, it appears that the benefits are not exclusive to a single type of exercise. Yoga [13], conventional aerobic training [15], combined aerobic and resistance programs [14, 16], and specific respiratory muscle training [17, 18] all showed positive effects on different outcome clusters. This suggests that the "best" intervention may be one that is feasible, enjoyable, and sustainable for the child. The success of yoga and breathing retraining points to the potential importance of the mind-body connection and the role of controlled breathing in managing asthma, a concept supported by research from Vempati et al. [24] on the efficacy of yoga in adult asthma. Conversely, the improvements in muscle strength and cardiorespiratory fitness from more conventional exercise programs [14, 15] are essential for overall physical health and combating the deconditioning that plagues this population.

The safety and feasibility of these interventions, even in relatively vulnerable populations, is an important takeaway. None of the included studies reported significant adverse events related to the exercise interventions, and studies like that of Bignall et al. [12] demonstrated that such programs can be successfully implemented in real-world settings like schools. This is a critical point for clinical translation, as it suggests that with proper screening and supervision, exercise is not only beneficial but also a safe and practical component of a comprehensive asthma management plan.

Limitations

This systematic review has several limitations that must be acknowledged. First, the number of included studies is relatively small (n=8), and the sample sizes within many of these trials were also modest, limiting the statistical power and the generalizability of the findings. Second, there was significant clinical and methodological heterogeneity among the studies, particularly in the type, frequency, intensity, and duration of the exercise interventions, as well as in the characteristics of the patient populations. This heterogeneity precluded a meta-analysis and makes it difficult to draw definitive conclusions about the optimal exercise prescription for pediatric asthma. Third, as noted in the risk of bias assessment, while most studies were of high quality, the inherent difficulty of blinding participants and personnel in exercise trials introduces a potential for performance bias. However, the objective nature of some outcomes, like VO₂max, and the consistency of subjective outcomes like QoL across studies, strengthen the validity of the findings. Finally, the follow-up durations in the included studies were generally short-term; thus, the long-term sustainability of the observed benefits remains unknown and warrants investigation in future studies with longer follow-up periods.

## Conclusions

This review provides robust evidence supporting the integration of aerobic exercise-based pulmonary rehabilitation into the standard care of children with bronchial asthma. While the effect on traditional spirometric measures like FEV₁ is variable, the interventions consistently demonstrate significant and clinically relevant benefits in the core domains of asthma management: symptom control, quality of life, and cardiorespiratory fitness. These benefits were observed across a range of exercise modalities, from yoga and aerobic training to inspiratory muscle strengthening. The findings advocate for a paradigm shift in which exercise is not viewed as a risk to be avoided but as a therapeutic tool to be prescribed and supervised. Future research should focus on standardizing optimal exercise protocols, understanding the underlying mechanisms of benefit beyond spirometry, and incorporating cost-effectiveness and feasibility analyses to evaluate the long-term sustainability and real-world implementation of these programs in pediatric settings.

## Appendices

Appendix 

**Table 5:** Detailed Search Strategy for each Database

Database	Search String
PubMed	("Bronchial Asthma"[MeSH Terms] OR "Asthma"[Title/Abstract]) AND ("Children"[MeSH Terms] OR "Pediatric"[Title/Abstract] OR "Adolescent"[Title/Abstract]) AND ("Aerobic Exercise"[MeSH Terms] OR "Exercise Training"[Title/Abstract] OR "Physical Activity"[Title/Abstract] OR "Exercise Therapy"[MeSH Terms]) AND ("Pulmonary Rehabilitation"[MeSH Terms] OR "Rehabilitation"[Title/Abstract])
Scopus	(TITLE-ABS-KEY("bronchial asthma" OR "asthma") AND TITLE-ABS-KEY("children" OR "pediatric" OR "adolescent") AND TITLE-ABS-KEY("aerobic exercise" OR "exercise training" OR "physical activity" OR "exercise therapy") AND TITLE-ABS-KEY("pulmonary rehabilitation" OR "rehabilitation"))
Embase	('bronchial asthma'/exp OR 'asthma':ti,ab) AND ('child'/exp OR 'pediatric':ti,ab OR 'adolescent':ti,ab) AND ('aerobic exercise'/exp OR 'exercise training':ti,ab OR 'physical activity':ti,ab OR 'exercise therapy'/exp) AND ('pulmonary rehabilitation'/exp OR 'rehabilitation':ti,ab)
Web of Science	TS=("bronchial asthma" OR "asthma") AND TS=("children" OR "pediatric" OR "adolescent") AND TS=("aerobic exercise" OR "exercise training" OR "physical activity" OR "exercise therapy") AND TS=("pulmonary rehabilitation" OR "rehabilitation")
ClinicalTrials (ClinicalTrials.gov)	("asthma" OR "bronchial asthma") AND ("children" OR "pediatric" OR "adolescent") AND ("aerobic exercise" OR "exercise training" OR "physical activity" OR "pulmonary rehabilitation" OR "rehabilitation")
